# The Utility of Serum Creatinine Kinase in Emergency Department Patients with Possible Substance-use Related Conditions

**DOI:** 10.5811/westjem.2020.5.46678

**Published:** 2020-09-04

**Authors:** Mohammad S. Alzahri

**Affiliations:** King Saud University, Department of Emergency Medicine, Riyadh, Saudi Arabia

## Abstract

**Introduction:**

Our goal was to assess the diagnostic utility and temporal kinetics of serum creatine kinase (CK) measurement as a predictor of acute kidney injury (AKI) in emergency department (ED) patients who present with possible substance-use related conditions.

**Methods:**

This was a retrospective chart review of ED patients with a urine drug screen (UDS) ordered and resulted between 2009–2013. Data was extracted electronically from EPIC Systems electronic health records, populated into a Microsoft Excel file, and includes demographics, chief complaint, vital signs, neuro-psychiatric physical examination findings, laboratory findings, psychiatric consult order time, ED medications given, orders, disposition and its time, and diagnosis.

**Results:**

Of 74,970 patients with an ED UDS, 22,101 (29%) had at least one CK measured. After inclusion and exclusion criteria, 2858 (13%) remained. Mean (standard deviation [SD]) age was 43.3 (12.5) years, 73% were male, 61% Black, 22% White, and 17% Hispanic. Mean (SD) ED length of stay was 10.4 (5.8) hours, and 56.7% were hospitalized. On average, CK was higher at 6–12 hours (p<0.001) and 12–18 hours (p=0.016) compared to 6 hours. CK was lower at 42–56 hours (p = 0.011), 72 hours (p<0.001), and over 72 hours (p<0.001), compared to 6 hours. Maximum CK was determined in those with >2 CK measures. We defined AKI risk as a creatinine of >1.4 milligrams per deciliter based on RIFLE criteria. AKI risk was calculated among those with at least two creatinine values in 522 patients. We identified five (1%) patients as having AKI risk. The odds of AKI risk were not associated with increase in CK over time.

**Conclusion:**

In 74,970 ED patients undergoing UDS testing for potential substance abuse, there was no identifiable CK level associated with AKI risk. In patients with possible substance-use conditions, CK continued to trend up even after six hours from door time and began to decrease after 42 hours. We found no value in repeated ED CK measures. Disposition should not be based solely on CK levels.

## INTRODUCTION

Behavioral emergencies are responsible for approximately 6% of all emergency department (ED) visits in the United States,^1^ where emergency physicians are frequently asked to exclude medical illnesses that may be causing or contributing to the patient’s acute psychiatric symptoms.^2^ While the policy of the American College of Emergency Physicians states that in adult ED patients with primary psychiatric complaints, diagnostic evaluation should be directed by the history and physical examination and that routine laboratory testing of all patients is of very low yield, considerable variation exists between physicians, departments, and institutions in what is generally considered necessary in the medical assessment of patients with acute psychiatric emergencies.^3^

Substance use is a common ED presentation, and alcohol, heroin, and cocaine have all been shown to cause rhabdomyolysis. Rhabdomyolysis causes about 7–10% of all cases of acute kidney injury (AKI) annually.^4^,^5^ Previous studies have shown that the incidence of AKI is higher among patients who have rhabdomyolysis as a consequence of illicit drug or alcohol use.^6^–^8^ The same study found a 3.4% mortality among this cohort of patients with substance use and AKI. Thus, the importance of identifying and treating potential rhabdomyolysis or AKI prior to medically clearing patients with substance use is evident.

A diagnosis of rhabdomyolysis is made by testing serum creatine kinase (CK) levels. The consensus definition has rather arbitrarily been chosen as five times the upper limit of normal, or approximately 1000 units per liter (U/L). More recently, proposed guidelines suggest that a diagnosis of rhabdomyolysis should be made only when the serum CK is higher than 50 times of the upper limit of normal or when CK elevation is accompanied by findings of AKI. This is because a mild to moderate elevation of serum CK above the normal reference range is expected in healthy adults after physical exertion. These mild to moderate elevations are often not clinically significant and do not require medical management, such as serial laboratory testing or intravenous (IV) hydration.^9^,^10^

Previously, weak correlations between the peak CK value and the incidence of AKI or peak serum creatinine have been reported.^4^ One study of 72 patients had shown that one-fourth of the patients with peak CK >10,000 U/L and positive drug screens for cocaine or heroin developed rhabdomyolysis-associated renal failure.^11^ Often, when AKI is seen at low CK levels around 5000 U/L, this occurs in the presence of several comorbidities such as sepsis, dehydration, and acidosis.^12^

It has been our experience that psychiatric facilities arbitrarily set low thresholds for serum CK above which they will not accept psychiatric patients in transfer until those levels normalize or show a decreasing trend. This practice typically leads to a delay in transfer of such patients to psychiatric facilities by way of serial laboratory testing, IV fluid therapy, and medical hospitalization—the benefit of which is not unequivocally proven.

Given the above, here we set out to investigate the diagnostic utility and temporal kinetics of serum CK measurement as a predictor of AKI in ED patients who present with possible substance-use related conditions. Some of the patients we labeled as AKI could have had acute renal failure. This will be further detailed in the “Methods” section. Our purpose was to assess the diagnostic utility and temporal kinetics of serum CK measurement as a predictor of AKI in ED patients who present with possible substance-use related conditions.

## METHODS

This was a retrospective cohort analysis of ED patients who presented to Ben Taub General Hospital (BTGH) and had a urine drug screen (UDS) ordered and resulted between 2009–2013. The UDS used was a quantitative, 10-panel immunoassay UDS that included amphetamine; barbiturate; benzodiazepines; cocaine; methadone; opiates; oxycodone/oxymorphone; phencyclidine; propoxyphene; and tetrahydrocannabinol, provided by American Screening Corp. BTGH is a Level 1 trauma center and the largest county hospital in Houston, Texas. It is the only hospital in Houston with a psychiatric ED that is open 24 hours a day. In 2009 the BTGH ED starting using the EPIC ED module ASAP (Epic Systems, Verona, WI), which allows for extraction of clinical data electronically. Data collection was completed by 2015, but statistical analysis was not done until 2018 as the author was matriculated in a full-time clinical training program.

**Population Health Research Capsule**

What do we already know about this issue?

There is conflicting information on how to use serum creatine kinase (CK) in evaluating patients with possible substance-use related conditions.

What was the research question?

*In patients with possible substance use*, *what level of CK is associated with acute kidney injury?*

What was the major finding of the study?

There was no value in repeated CK measures in the emergency department (ED). Disposition should not be based solely on CK level.

How does this improve population health?

This finding will help decrease unnecessary testing, lowering ED length of stay, and consequently ED waiting time.

Data was extracted electronically from EPIC, populated into a Microsoft Excel (Microsoft Corporation, Redmond, WA) file, and includes demographics, chief complaints, vital signs, neuro-psychiatric physical examination findings, laboratory findings, psychiatric consult order time, ED medications given, orders, disposition and its time, and diagnosis. Inclusion criteria were either a positive UDS or serum ethanol >0.08 grams per deciliter (g/dL), with a chief complaint, diagnosis, or physical finding of intoxication, agitation, drug use, or confusion. Exclusion criteria included conditions that may alter CK results, including a history of chronic kidney disease, age >65 years, or a temperature >102 degrees Fahrenheit at any time.

### Statistical Analysis

Patient characteristics were summarized by frequency with percentage and mean with standard deviation. AKI risk was defined as a creatinine of > 1.4 times baseline creatinine, as defined by RIFLE (Risk, Injury, Failure, Loss of kidney function, and End-stage kidney disease) classification. It is commonly labeled simply as AKI. We used the RIFLE term “AKI risk.”^13^ We labeled all patients with creatinine >1.4 × baseline as AKI risk with no subset analysis for patients with renal failure. As a result, these patients can be more accurately labeled as “at least AKI risk.” CK and creatinine were then summarized over time. A mixed model linear regression with discrete residuals was used to assess log transformed CK over time. Note that due to the skewed nature of the data, CK was log transformed.

This study was approved by the Baylor College of Medicine (BCM) Institutional Review Board. This approval was maintained until data analysis was complete by 2018. Patient confidentiality was maintained. All data were stored on a password-protected computer accessible only to study investigators. The password-protected computers were managed and maintained by the BCM information technology department. Patients were not directly contacted. The original file included the patient’s CSN# (account number) as well as a running counter. The file was kept in the principal investigator (PI)’s password-protected BCM computer located in the PI’s .locked office. The PI de-identified the file (deleted CSN/medical record [MR]# columns) and saved the PHI redacted Excel file as a new password-protected file. The PHI (MR#/CSN) were deleted from the dataset before analysis.

## RESULTS

There were 74,970 patients in the dataset, 52,869 with no CK measures and 22,101 (29%) with at least one CK measure. A total of 4272 patients had a chief complaint, diagnosis, or physical finding of intoxication, agitation or drug use, and 3085 of them had positive UDL or serum ethanol >0.08 g/dL. Eighty-eight patients were older than 65 years old; 72 of the remaining 2997 patients had history of chronic kidney disease; and 67 out of the remaining 2925 patients had a temperature more than 102º F at any time. Therefore, after applying the rest of the inclusion and exclusion criteria, 2858 (13%) patients remained. Mean age was 43.3 (standard deviation 12.5) years, 73% were male, 61% Black, 22% White, and 17% Hispanic.

**Figure 1:**
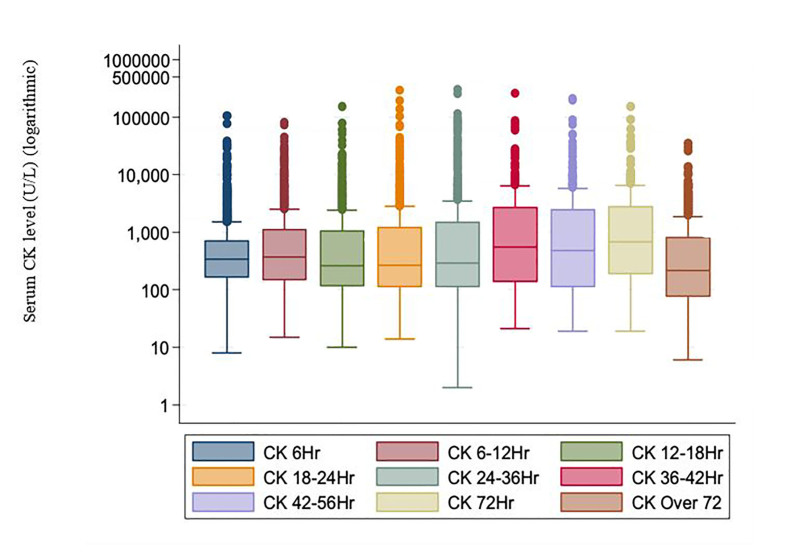
Serum creatine kinase levels over time in patients presenting to the emergency department with substance use. *CK*, creatine kinase; *ED*, emergency department.

On average, CK was higher at 6–12 hours (p<0.001) and 12–18 hours (p = 0.016) compared to 6 hours. Additionally, CK was lower at 42–56 hours (p=0.011), 72 hours (p<0.001), and over 72 hours (p<0.001), compared to 6 hours. CK geometric mean over time is shown in Figure 1. The geometric mean is defined as the nth root of the product of n numbers for each time interval. Note that the y-axis of the figure is on the log scale. Fewer than 10 measures had a CK of zero that were dropped for the figure (since log of zero cannot be calculated).

**Figure 2:**
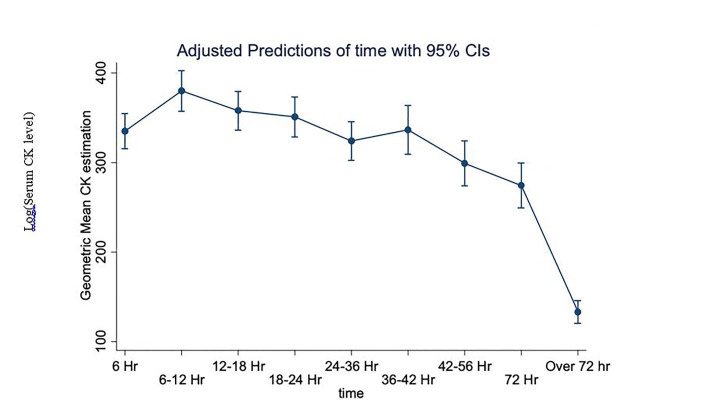
Log transformed serum creatine kinase level over time in patients presenting to the emergency department with substance use. The average CK over time is shown with 95% confidence intervals in Figure 2. The geometric mean for CK is 335 U/L at 6 hrs, 380 U/L at 6–12 hrs, 358 U/L at 12–18 hrs, 351 U/L at 18–24 hrs, 324 U/L at 24–36 hrs, 336 U/L at 36–42 hrs, 299 U/L at 42–56 hrs, 274 U/L at 72 hrs, and 133 U/L at over 72 hrs. *CK*, creatine kinase; *ED*, emergency department.

On average, CK is higher at 6–12 hours (p<0.001), and 12–18 hours (p = 0.016) compared to 6 hours. When compared to 6 hours on average, CK is lower at 42–56 hours (p = 0.011), 72 hours (p<0.001), and over 72 hours (p<0.001). This is available in supplemental materials as Table 1: The mixed model results for log CK over time. The average CK over time is shown with 95% confidence intervals in Figure 2. A figure of CK over time for those with at least three CK measures is available in supplemental materials. The geometric mean for CK is 335 U/L at 6 hours; 380 U/L at 6–12 hours; 358 U/L at 12–18 hours; 351 U/L at 18–24 hours; 324 U/L at 24–36 hours; 336 U/L at 36–42 hours; 299 U/L at 42–56 hours; 274 U/L at 72 hours; and 133 U/L at over 72 hours.

When looking at maximum CK (Table 1), we only used patients with at least three CK measures, which was 888 U/L. AKI risk was calculated among those with at least two creatinine values, or 522 patients. There were five (1%) patients identified as having AKI risk. The odds of AKI risk were not associated with increase in CK over time.

Table 2 shows the logistic regression results for AKI risk. Note that each row is a separate model for logistic regression and that times 6–12 hours and 72 hours could not converge. When looking at the full population, mean (SD) ED length of stay was 10.4 (5.8) hours and 56.7% were hospitalized (Table 3). Admissions were to either medical or psychiatric wards within the hospital. All the transferred patients were transferred to another psychiatric facility when no bed was available in the psychiatric ward.

## DISCUSSION

Most labs set upper normal limit of serum CK as 198 U/L.^4^ Although a variety of conditions can contribute to serum CK above that level, most of them are not clinically significant. The clinical significance of rhabdomyolysis lies when there is associated acute renal failure and, subsequently, electrolyte disturbance.^14^ Ruling out clinically significant rhabdomyolysis in the ED often can be challenging. Incidence of acute renal failure secondary to rhabdomyolysis varies according to the cause of the disease and can range between 1–45%.^4^ While limited studies reported the incidence of renal failure in patients with substance use, most were done in a chronic substance use setting. These studies were also limited by sample size, which ranged between 16 to 716 patients.^15^–^18^ Moreover, some results contradicted each other. While most studies report AKI incidence of less than 5%, a study by Akmal et al reported 40% of acute renal failure from rhabdoyolysis cause by phencyclidine.^19^

Using the electronic health record, we were able to extract data for a relatively large sample size, or 74,970 patients who had UDS results and 22,101 (29%) who had at least one CK measured. With this large sample size, we were able to better assess early temporal kinetics of serum CK measurement and its diagnostic utility in ED.

The finding that serum CK levels continued to trend up and did not fall significantly below arrival measurements until after 42 hours supports that there is limited value in repeated measurements in the ED and a patient’s disposition should not be based solely on CK levels. Accordingly, when patients need to be transferred to a psychiatric facility, the accepting facility may not benefit from a repeated serum CK level and decision whether patient can be medically cleared should be based on other factors. We recommend discussing this finding with psychiatric departments’ decision-makers through interdepartmental meetings or in the form of a letter to psychiatric hospitals’ medical directors.

As in previous studies,^20^ only 1% of patients had AKI risk. It is worth noting that many of these patients were brought in by emergency medical services, where prehospital care could have been started that may have included IV fluid administration and, subsequently, low incidence of AKI risk. Although a similar incidence was reported in prior studies, our extracted data did not indicate whether such prehospital intervention was done.

Unlike some prior studies, we did not find an identifiable CK level associated with AKI, which could have been attributed to the small number of this subset group. Nevertheless, the finding that the odds of AKI are not associated with an increase in CK over time was limited by the small sample of this subset group analysis.

## LIMITATIONS

One of the limitations in this retrospective cohort study was that patients included in the study only had possible substance abuse. Inclusion criteria was either legally intoxicated with alcohol, as per the State of Texas serum alcohol level > 0.08, or had positive substance with a UDS and deemed intoxicated by the clinician (physician, physician assistant or nurse practitioner). Due to the fact that a positive predictive value of current UDS testing can be very low for some substances,^21^ only patients who were deemed intoxicated by the clinician were included in the study, for both alcohol and UDS. In patients who were included, the clinician indicated in the chart (either as patient self-reported chief complaint, clinical impression diagnosed as an International Classification of Diseases, Editions 9/10 diagnosis, or physical exam finding – usually predefined check boxes – that a patient had one of the following: intoxication, agitation, drug use, or confusion. While exclusion criteria included conditions that could affect CK,^4^ these are predefined boxes in the history section of the ED chart that are often skipped by emergency clinicians. Another limitation is that patients who reported drug use by history and had negative UDS were not included in the study. Some of these patients could have had false negative results.

We defined AKI risk according to RIFLE criteria, which classifies elevation of serum creatinine >2x baseline as renal failure. We labeled all patients with creatinine >1.4x baseline as AKI risk with no subset analysis for patients with renal failure. This was due to the small sample size in this group. Although we excluded patients with end stage renal disease, we did not included glomerular filtration rate or urine output, both of which are significant factors of RIFLE.^13^ However, it would have been helpful to have carried out a subset analysis to each substance and alcohol separately, as some prior studies reported varying level of morbidity in certain substances.^22^ Further, it is possible that some substances had a diluting effect, as a previously mentioned study by Akmal et al. reported a 40% incidence of acute renal failure from rhabdomyolysis in the setting of phencyclidine use.^18^ Another limitation was the fact that we did not perform prior power analysis. Therefore, it is possible that this study was not powered enough to detect an association between AKI risk and an increase in CK over time, if there was any.

Although we did not derive valuable information in regard to ED length of stay, one may notice the relatively long average ED stay. In this county/teaching hospital, both waiting time and boarding time are relatively greater than other community hospitals. This is more evident in this patient population. Due to the fact that BTGH is the only psychiatric ED open 24 hours in Houston, it is possible that many of these patients needed inpatient psychiatric admission or transfer to another Harris county psychiatric facility, both of which can further delay disposition.

## CONCLUSION

In this population of patients presenting to the ED with substance use, we found that there was no identifiable CK level associated with increased AKI risk. We found that CK levels continued to trend up and did not fall significantly below arrival measurements until after 42 hours. Our findings support that there is limited value in repeated ED CK measurements, and disposition should not be based solely on CK levels. Further research is needed to expand understanding of the risk relationship between AKI risk, creatinine, and CK levels with the potential for more judicious ordering of diagnostic lab tests.

## Supplementary Information



## Figures and Tables

**Table 1:** Time interval associated with maximum serum creatine kinase (CK) level in patients with 3 CK measurements taken, n = 888.

Time interval of Measurement (hrs)	Frequency (n) of Maximum CK	%
<6	218	24.6
6 – 12	168	18.9
12 – 18	153	17.2
18 – 24	124	14.0
24 – 36	96	10.8
36 – 42	51	5.7
42 – 56	31	3.5
72	22	2.5
>72	25	2.8

In patients presenting to the ED with at least 3 serum CK measurements, the maximum CK was most frequently seen at the initial time interval prior to 6 hours. The frequency in which the maximum CK level was seen decreased as the time after 6 hours increased.

*CK*, creatine kinase; *hrs*, hours*; ED*, emergency department.

**Table 2:** Univariate logistic regression for acute kidney injury based on 500-unit Increase in serum creatine kinase level.

Time interval of measurement (hrs)	Odds ratio	95% CI	N
<6	0.01	0.00–0.52	234
12 – 18	0.71	0.47–1.07	173
18 – 24	0.86	0.64–1.17	167
24 – 36	1.00	1.00–1.01	168
36 – 42	0.92	0.89–0.96	84
42 – 56	1.01	1.00–1.01	101
>72	1.06	0.99–1.12	110
Max	1.00	1.00–1.01	205

Note that each row is a separate model for logistic regression and that times 6–12 hrs and 72 hrs could not converge. The odds of acute ikidney injury (AKI) risk are decreased as creatine kinase (CK) increases for 6 hrs (p=0.021) and 36–24 hrs (p<0.001). No other CK measures are statistically associated with AKI.

*hrs*, hours; *CI*, confidence interval; *ED*, emergency department.

**Table 3:** Creatine kinase measurement status and final disposition effect on mean ED length of stay, n = 74,970.

Patient characterisitics	N	Mean ED LOS in hours (SD)	P-value
CK measurement			
No	52,818	10.7 (6.0)	reference
Yes	22,101	10.4 (5.8)	<0.001
Disposition			
Home	32,438	10.9 (5.7)	reference
Other	42,532	10.5 (6.1)	<0.001

When looking at the full initial patient population presenting to the ED with a chief complaint of intoxication, agitation, drug use, or confusion, and either a positive urine drug screen or serum ethanol > 0.08 g/dl, the mean ED LOS was higher in those patients who had no CK measurements done (p < .001) and those patients who were discharged home (p < .001).

*CK*, creatine kinase*; ED*, emergency department*; LOS*, length of stay; SD, standard deviation.
