# Supplementary material for: The Utility of Serum Creatinine Kinase in Emergency Department Patients with Possible Substance-use Related Conditions
Source: West J Emerg Med. 2020 Sep 4;21(5):1195–200. doi: 10.5811/westjem.2020.5.46678 (PMC7514414; doi:10.5811/westjem.2020.5.46678)
Supplement: Supplementary file 1 [file wjem-21-1195-s001.docx]

**Supplemental Tables.**

**Table 1.** Mixed-model of log of serum creatine kinase measurements taken over time.

| Time interval (hrs) | Coefficient | [95% Confidence interval] | | P-value |
| --- | --- | --- | --- | --- |
| <6  6 – 12  12 – 18  18 – 24  24 – 36  36 – 42  42 – 56  72  >72 | Reference  0.13  0.07  0.05  -0.03  0.00  -0.11  -0.20  -0.92  5.81 | 0.08  0.01  -0.02  -0.10  -0.08  -0.20  -0.30  -1.02  5.76 | 0.17  0.12  0.11  0.03  0.09  -0.03  -0.10  -0.82  5.87 | < 0.001  0.016  0.140  0.316  0.931  0.011  < 0.001  < 0.001  < 0.001 |

The parameter estimates for a linear mixed model. We added this below the table: The only variable in the model is time (discrete). This is called a fixed effect. The time categories were created when data was extracted from EPIC. The random effect is because this is repeated measures. We had to account for the correlation between measures observed on the same patient. This impacts the estimates of standard errors (and p-values).

This shows the mixed model results for log CK over time. On average CK is higher at 6-12 hrs (p<0.001), 12-18 hrs (p=0.016) vs. 6 hrs. When compared to 6 hrs, on average CK is lower at 42-56 hrs (p=0.011), 72 hrs (p<0.001), and over 72 hrs (p<0.001).


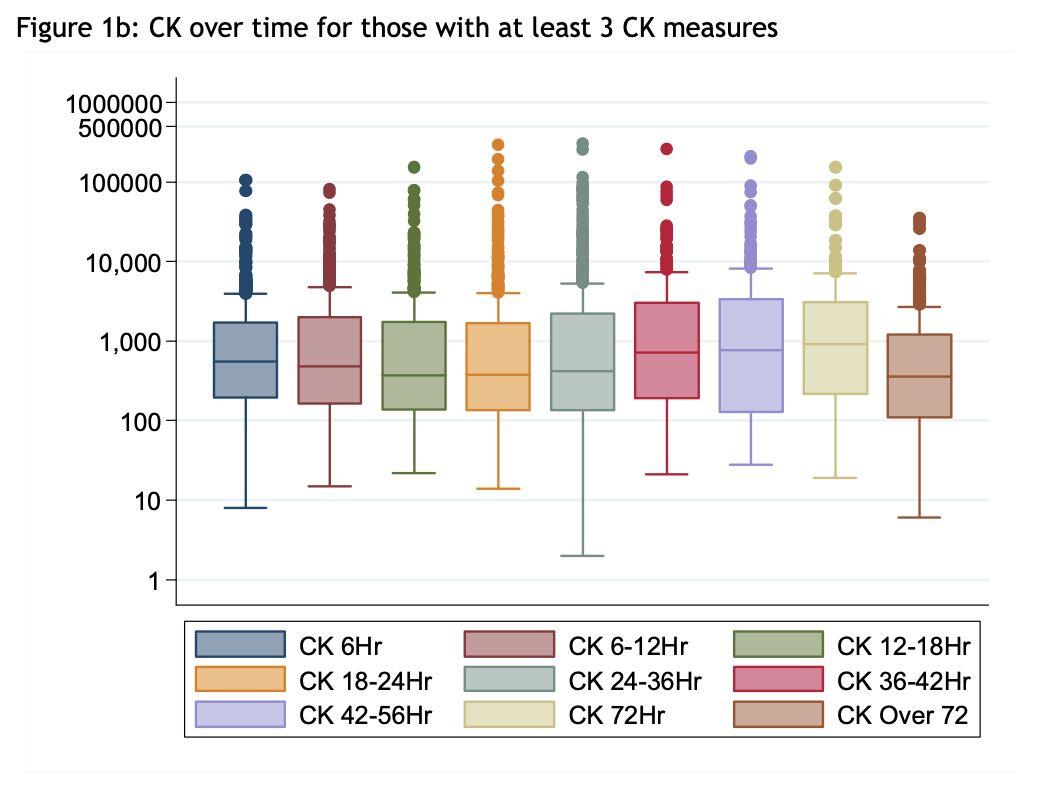
*CK,* creatine kinase.

**Figure 1.** Creatine kinase (CK) over time for those with at least three CK measures.

*CK,* creatine kinase.


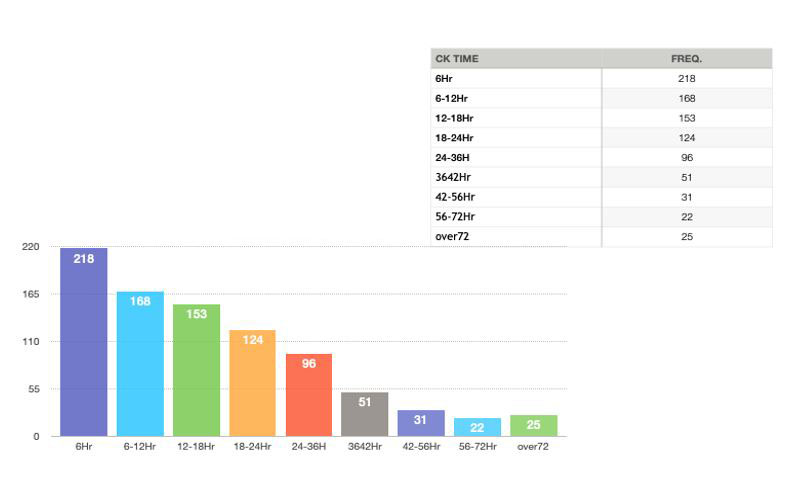


**Figure 2.** Creatine kinase measures/times frequencies.
